# Harnessing Intranasal Delivery Systems of Sumatriptan for the Treatment of Migraine

**DOI:** 10.1155/2022/3692065

**Published:** 2022-01-15

**Authors:** Sara Assadpour, Mohammad Reza Shiran, Peyman Asadi, Javad Akhtari, Amirhossein Sahebkar

**Affiliations:** ^1^Molecular and Cell Biology Research Center, Faculty of Medicine, Mazandaran University of Medical Sciences, Sari, Iran; ^2^Department of Pharmacology, Faculty of Medicine, Mazandaran University of Medical Sciences, Sari, Iran; ^3^Department of Medical Nanotechnology, School of Advanced Technologies in Medicine, Mazandaran University of Medical Sciences, Sari, Iran; ^4^Toxoplasmosis Research Center, Communicable Diseases Institute, Faculty of Medicine, Mazandaran University of Medical Sciences, Sari, Iran; ^5^Applied Biomedical Research Center, Mashhad University of Medical Sciences, Mashhad, Iran; ^6^Biotechnology Research Center, Pharmaceutical Technology Institute, Mashhad University of Medical Sciences, Mashhad, Iran; ^7^Department of Biotechnology, School of Pharmacy, Mashhad University of Medical Sciences, Mashhad, Iran

## Abstract

Sumatriptan (ST) is a commonly prescribed drug for treating migraine. The efficiency of several routes of ST administration has been investigated. Recently, the intranasal route with different delivery systems has gained interest owing to its fast-acting and effectiveness. The present study is aimed at reviewing the available studies on novel delivery systems for intranasal ST administration. The oral route of ST administration is common but complicated with some problems. Gastroparesis in patients with migraine may reduce the absorption and effectiveness of ST upon oral use. Furthermore, the gastrointestinal (GI) system and hepatic metabolism can alter the pharmacokinetics and clinical effects of ST. The bioavailability of conventional nasal liquids is low due to the deposition of a large fraction of the delivered dose of a drug in the nasal cavity. Several delivery systems have been utilized in a wide range of preclinical and clinical studies to enhance the bioavailability of ST. The beneficial effects of the dry nasal powder of ST (AVP-825) have been proven in clinical studies. Moreover, other delivery systems based on microemulsions, microspheres, and nanoparticles have been introduced, and their higher bioavailability and efficacy were demonstrated in preclinical studies. Based on the extant findings, harnessing novel delivery systems can improve the bioavailability of ST and enhance its effectiveness against migraine attacks. However, further clinical studies are needed to approve the safety and efficacy of employing such systems in humans.

## 1. Introduction

Migraine, one of the most common neurological disorders globally, has been ranked as the second cause of disability among young and middle-aged individuals [1, 2]. This neurovascular dysfunction is featured by recurrent episodes of disabling unilateral headache with sensitivity to movement, visual, auditory, and dysfunction in the autonomic nervous system. In some cases, neurological aura symptoms have been reported [3]. Females are affected predominantly (3 : 1), with poor quality of life during the most productive age [2].

Migraine is the sixth most prevalent disabling illness, affecting 15.1% of the population around the world [4]. This disorder involves intense, recurrent headaches and other associated unpleasant symptoms [5]. As a long-term disease, different age groups can be affected by migraine. Although the prevalence of migraine varies between children in a wide pediatric age range, it has an insignificant variation between girls and boys [6]. In adults, migraine in women is more prevalent (12-17%) than men (4-6%) [7]. Migraine is categorized into episodic or chronic forms, accompanied by an aura. An aura is a perceptual disturbance experienced by some with epilepsy or migraine. Migraine headaches usually begin with activating sensory afferent fibers from the ophthalmic branch of the trigeminal nerve.

Since the cerebral cortex of many patients with migraine is highly sensitive, abnormal cortical activity leads to cortical spreading depression (CSD), which causes the release of various mediators into the extracellular fluid. Changes in cell fluid lead to activation of the trigeminal nerve (TGN). Upon activation of TGN, neuropeptides are released by trigeminal ganglion neurons, leading to neurogenic inflammation in the dura mater. In other words, meningeal dilation and cerebral endothelial dysfunction lead to the destruction of mast cells and the release of proinflammatory materials. Abnormal and persistent stimulation of the trigeminal nerve can accelerate central sensitivity. Finally, the data suggest that activation of TGN afferents effectively initiates migraine pain and central sensitivity [8].

Currently, the therapies for migraine are primarily nonspecific, described by poor patient compliance. The successful medications for acute migraine are nonsteroidal anti-inflammatory (NSAID) drugs, ergot alkaloids, and triptans (serotonin hydroxytryptamine (5-HT)_1B/1D/IF_ receptor agonists) [9, 10]. Triptans are specific antimigraine drugs that effectively can relieve migraine pain. As a first-line treatment, triptans are used for moderate-to-severe migraine headaches. However, their application is usually limited due to adverse effects, time-and frequency-restricted use, and the risk of emerging drug overuse headache [11].

In this class, sumatriptan (ST), the most commonly prescribed drug, was approved by the US FDA for migraine attacks in 1992 [12]. ST administration reveals the strongest antiemetic function which can benefit migraine-related *nausea* [13]. The safety and effectiveness of various routes of ST (e.g., oral, intranasal, transdermal, subcutaneous, and rectal) have been investigated in several clinical trials, and related formulations are in use [14–19]. Some reports address cardiac adverse reactions related to sumatriptan as an antimigraine drug. Adverse reactions such as paraesthesiae, dizziness, and chest pain have been reported [20].

While the oral route is the most frequent, variability in gastric emptying during the migraine attack and subsequently delayed absorption may cause inconsistent efficiency, such as the delayed onset of action and decreased magnitude of relief [21]. Oral medicines are easy to use but have some problems, mainly in patients with migraine. Furthermore, gastroparesis (delayed gastric emptying) may arise during or between migraine attacks and seems to decrease the consistency, absorption, and efficiency of oral treatments. Gastrointestinal and hepatic first-pass metabolism may change the pharmacokinetics and clinical properties of oral medications. Significantly, oral ST reduced headache relief in one-third of cases in clinical trials [22]. Likewise, sumatriptan succinate (SS) (the reaction of ST with one equivalent of succinic acid) is not completely absorbed from the gastrointestinal tract, as it is highly metabolized. Thus, oral SS also has low bioavailability (~15%) [13]. The amplified lipophilicity of ST to enhance its bioavailability negatively affected its physicochemical features and solubility levels, which were essential for improving pharmaceutical formulations [23].

To overcome oral route limitations, the intranasal route (nasal spray) has been introduced to improve the consistency and speed of absorption of the medication and prevent the complications related to self-administration injection [24]. Sumatriptan poorly penetrates the central nervous system [25]. A nasal cavity with a highly vascular membrane can promote rapid absorption of metabolized drugs to the central nervous system (CNS) [26]. It has been proven that intranasal drug delivery (IN) is superior to other routes for bypassing the blood-brain barrier (BBB) and efficient brain targeting [27]. Therefore, the transfer of drugs to the brain can be improved by IN enhancement. However, some limitations, such as mucociliary clearance, reduce drug absorption in the nasal cavity [28, 29]. Moreover, different nasal delivery approaches of triptans have been reported to be fast-acting and effective for treating migraine [22]. In recent studies, there are significant indications that sumatriptan can cross the BBB. The CNS adverse events of sumatriptan in patients with migraine and regular volunteers also indicate a more general effect of sumatriptan on CNS, indicating that the drug can cross the BBB. It has been discussed whether a defect in the BBB during migraine attacks could be responsible for a possible central effect of sumatriptan in migraine. It was reported that there is no need for a breakdown in the BBB to occur for explaining a possible central CNS effect of sumatriptan [30].

Recently, using new strategies for increasing ST bioavailability has attracted more attention in clinical researches [26]. All intranasal complexes of ST (substituted and unsubstituted) have been demonstrated an improvement in pharmacokinetic features after nasal application due to higher bioavailability and solubility [13]. The available intranasal approaches use standard single-dose nasal spray pumps that characteristically deposit a substantial fraction of dose along the floor of the nasal cavity [31].

In this review, we tried to summarize ST's underlying mechanism of action and the effects of its intranasal novel formulations on migraine. This review highlights the current available intranasal delivery approaches for migraine treatment, including the dry nasal powder of ST (AVP-825), microemulsions, microspheres, and nanoparticles.

## 2. Migraine and Its Underlying Mechanisms

Migraine auras are due to the involvement of the brain-specific areas, which determine the aura symptoms. Therefore, if the visual area is affected, the aura will consist of visual symptoms, while if a sensory one, then sensory symptoms will occur [32]. As a complex and multidimensional condition, migraine is affected by genetic and environmental parameters [33]. Studies demonstrated that genetic factors play a role in the etiology of migraine. The genetic variability is additive, with a negligible contribution of nonadditive genetic effects. The genetic contributions were similar in women and men despite a higher prevalence in women. Environmental factors are equally important, and these factors are individual to the migraineurs [34]. Generally, it has been confirmed that the trigeminovascular system, as a possible underlying mechanism of migraine, is activated via proinflammatory factors or oxidative stress [35].

Over the past decade, abundant evidence accumulated from animal and human data has shifted the focus from blood vessels toward a more integrated theory that implicates both vascular and neuronal components.

In particular, it has become increasingly evident that the activation of meningeal afferents, neuropeptide release, and neurogenic inflammation plays a pivotal role in the generation of pain in migraine headaches [36]. According to the recent theory, migraine symptoms may be related to repeated migraine attacks that target the central pain signaling pathways via induction of chronic sensitization. One crucial pathway influenced by central sensitization is the stimulation of the trigeminal nucleus [37]. Moreover, the activation of microglia and secretion of inflammatory mediators modulate central sensitization [38].

Among several inflammatory factors, interleukin-1*β* (IL-1*β*) were significantly increased during migraine attacks and interictal state (the period between episodes), which led to enhanced interactions between neurons and satellite cells [39]. The intracellular maturation of IL-1*β* is mediated by NOD-like receptor protein-3 (NLRP-3) inflammasome, an innate immune complex participating in the underlying pathologic mechanisms of neurological diseases [40]. Recently, the prominent role of NLRP3 inflammasome activation in central sensitization has been proven [37], suggesting that targeting this complex may be a suitable approach for managing migraine attacks.

## 3. Mechanism of ST

Triggering the trigeminovascular pathway leads to vasodilation of the meninges, central sensitization, and inflammation and contributes to the head pain phase of a migraine attack [41]. The elongated activation is followed by the sensitization of the trigeminovascular system in response to short-term exposure of the dura to a mixture of inflammatory mediators, including prostaglandin, bradykinin, serotonin, and histamine [37]. It has been shown that these mediators may stimulate visceral and somatic nociceptors in the rat, with higher algetic potency in humans [37]. High levels of serotonin 5-HT_1B/D_ receptors have been observed on the cranial vessels and the trigeminal nerve. Serotonin 5-HT_1B/D_ receptor agonists, specific antimigraine drugs, particularly triptans, are effective in the treatment of migraine attacks by targeting the trigeminovascular system and reestablishing the normal serum concentrations of calcitonin gene-related peptide (CGRP) [42].

Triptans can stimulate vasoconstriction and reduce neurogenic inflammation by diminishing the production of CGRP and substance P (a regulator of dura mater sterile inflammation) [13]. Besides, it was demonstrated that ST suppressed the electrophysiological action of acid-sensing ion channels located on the trigeminal ganglia via a cAMP-related pathway and 5-HT_1D_ receptor subtype in a dose-dependent manner in the rat [43]. ST can inhibit prooxidative enzymes such as inducible nitric oxide synthase (iNOS) and lipid peroxidase [44]. Furthermore, ST is a powerful antioxidant compound that can directly scavenge free radicals like superoxide and hydroxyl radicals [45, 46]. Hence, ST may decrease malondialdehyde (MDA) concentrations directly via decreasing lipid peroxidation and/or indirectly by suppressing free radical release [47]. Additionally, it has been reported that ST shows neuroprotective properties via reducing inflammatory mediators such as caspase-3, IL-1*β*, and tumor necrosis factor *α* (TNF*α*) in the dorsal ganglion of animals with vincristine-induced peripheral neuropathy. In addition to the agonistic features of serotonin receptors, ST may exert therapeutic effects on migraine via anti-inflammatory and antioxidant properties [48].

## 4. Different Intranasal Delivery Systems of ST

**Figure 1:**
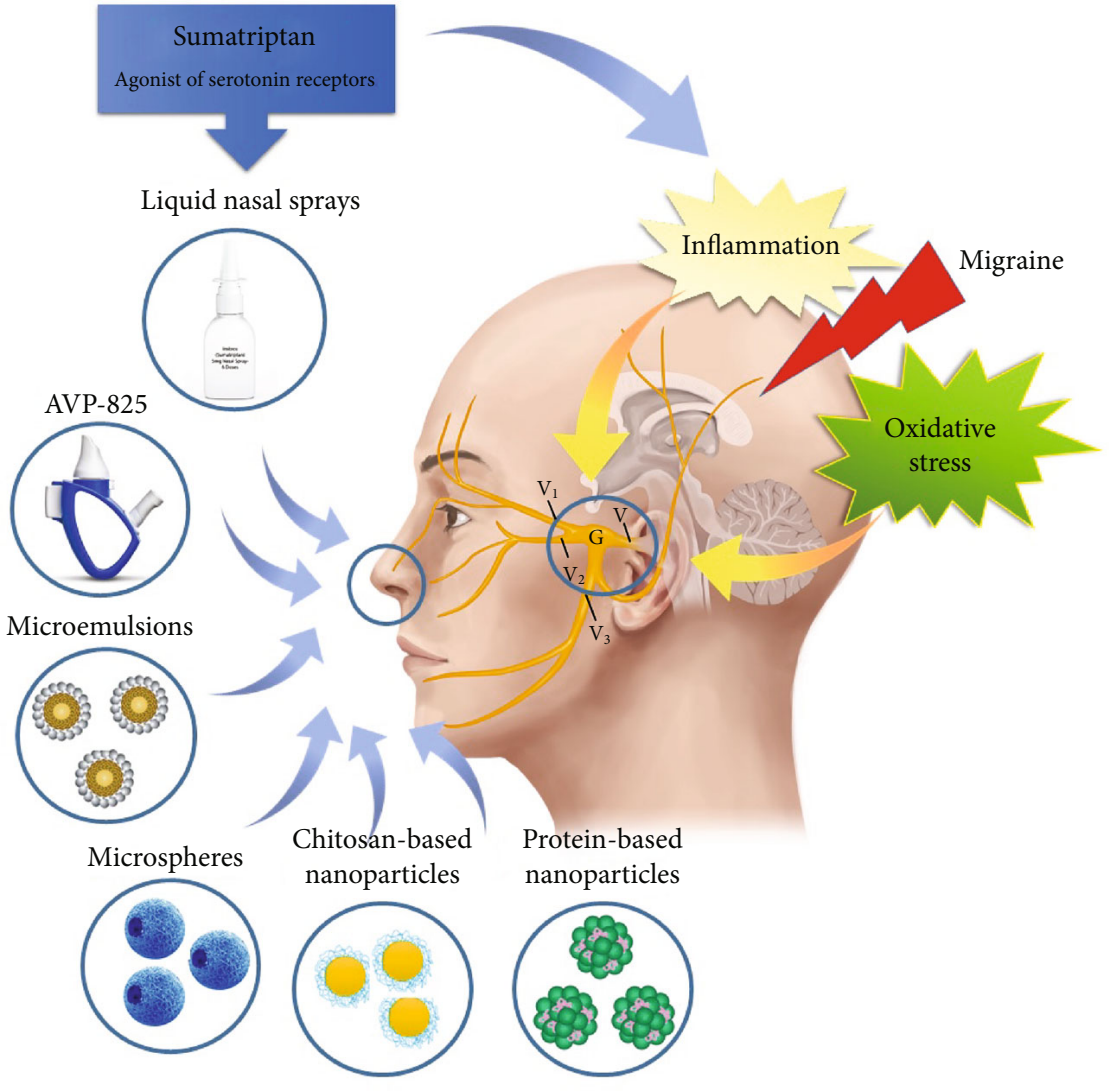
Different intranasal delivery systems of sumatriptan for the treatment of migraine. Different forms of intranasal delivery of sumatriptan for the treatment of migraine.

Although ST has been available for approximately 30 years, an efficient dosage form capable of drug delivery to the brain by nasal route has not yet been fabricated. The passage of orally administered ST to the brain is controlled by BBB, composed of capillary endothelial cells, astrocytes, and pericytes. The lack of BBB in the olfactory bulb region is one of the essential factors that promote the entry of drugs into the brain. IN administration of small-molecule drugs may allow them to bypass the BBB by crossing through the olfactory bulb [49]. As the intranasal route is an accepted route to improve nose-to-brain transport, several drug delivery systems of ST have been developed. In this part, different properties of these systems were discussed according to the available preclinical and clinical studies (summarized in Figure 1).

### 4.1. ST and Liquid Nasal Sprays

The nasal spray device with a liquid formulation of ST has been established due to the faster onset of relief and fewer side effects than injection forms. Despite the benefits, the reduced actual intranasal delivery due to the deposition of an extensive amount of the delivered dose of ST in the part of the nasal cavity was proven for these conventional liquid nasal sprays, and it is already an approved drug for use [50]. Besides, imaging techniques revealed that deposition of the drug from the intranasal spray pump principally occurs in the anterior region of the nasal valve and on the interior floor. Therefore, a limited part of the liquid can be presented in the posterior nasal cavity to be absorbed [51]. The anterior portion consists of the nonciliated squamous epithelium that has limited activity in the absorption of the medication. Notably, following nasal spray administration, a large portion of the remaining medication seems to enter the pharynx eventually and is swallowed [51]. Therefore, after swallowing, this amount of drug has the same procedure as the oral route with reduced efficacy due to gastrointestinal exposure [16]. Moreover, after utilizing these nasal sprays, the bitter taste of ST is usually recorded due to the exposure of liquid medication to the bitter-sensing taste buds located at the base of the tongue [22]. Consequently, the efficacy of conventional nasal spray decreases due to deposition or gastrointestinal exposure of a large portion of the liquid drug.

### 4.2. Dry Nasal Powder of ST (AVP-825)

Dry nasal powder of ST (AVP-825, ONZETRA® Xsail®) is a drug delivery device containing ST powder, developed for the acute management of migraine (with/without aura), which provides a low-dose sumatriptan powder to the out-of-access but very vascular mucosa beyond the nasal cavity. It has been developed based on the particular properties of nasal physiology and anatomy to reduce the limitations of liquid sprays. The device's adaptation to the anatomy of the nasal cavity, including the nasal valve opening and soft palate closure and the richly vascular mucosa of the upper posterior nose, leads to the deep deposition of ST into the cavity during delivery of ST powder. In contrast, it avoids ST deposition in the oropharynx or lungs [52]. This method can improve intranasal delivery to enter more efficient doses to the upper posterior nasal mucosa [53]. The low dose of ST (22 mg) in the dry powder formulation of the AVP-825 system has several potential benefits compared to the liquid formulation, such as the reduced need for preservatives, higher adhesion to the absorptive nasal mucosal surfaces, and superior stability [53]. Results from clinical PK and Phase II and III trials are consistent with fast sumatriptan absorption following AVP-825 administration and demonstrate that AVP-825 can improve early migraine pain, disability, and associated symptoms and favorable tolerability with minimal triptan-related adverse effects [53]. In several clinical studies, the efficiency and safety of AVP-825 have been evaluated. About the safety evaluation of AVP-825, it has been reported that in all three AVP-825 controlled trials, no serious adverse events were observed. The most common adverse events were mild and limited to the administration site. The safety findings of the comparative efficacy trials (Phase III COMPASS) have been consistent with those of the placebo-controlled trials. In addition, systemic treatment-emergent adverse events for AVP-825 were similar to oral sumatriptan 100 mg [54].

In a comparative study of AVP-825 versus ST tablets, reduced treatment-emergent nausea was recorded for AVP-825 [55]. In the following, the results of the COMPASS study (a double-blind, randomized, multicenter, comparative study with two-week duration) showed that treatment of migraine with AVP-825 was related to the reduced pain disability and intensity (10-90 min) and higher within-person consistency through multiple attacks (45-120 min) compared to oral ST. These results may reveal the fast and reliable absorption of ST and a rapid onset of therapeutic effects in the AVP-825 group [56]. The findings of the same study also proved that earlier and more consistent improvements in headache and other migraine-related manifestations were provided by oral ST, emphasizing the clinical benefits of this novel intranasal delivery system [57]. In another study from the COMPASS project, AVP-825 had more promising nausea consequences. Treatment with AVP-825 resulted in significantly quicker decreased odds of nausea during the 30 min-2 h following treatment and reduced rates of overall nausea after one hour of administration and reduced risk of emergent nausea (TEN) compared to oral ST, highlighting the effectiveness of AVP-825 in the management of nausea in the acute treatment of migraine [58]. Since the absorption of nasally delivered sumatriptan powder is independent of the GI system, AVP-825 is likely to be helpful in acute migraine with dysfunction of GI [53]. Another study from a similar project (COMPASS) conducted among 259 study participants has indicated faster reductions in migraine pain intensity and disability. The reduction was reported to be initiated 10 minutes postdose and lasted for the first 30 minutes (migraine pain intensity) and 45 minutes for migraine-related disability. The results demonstrated a lower overall pain intensity and disability that remained for the first 2 hours after therapy with AVP-825 compared with 100 mg oral sumatriptan [59]. AVP-825 may have the potential to be used at all phases of a migraine attack. The COMPASS study implies that this formula provides higher efficacy at early time points vs. tablets. Despite confirming the beneficial effects of AVP-825, a randomized clinical trial is under process (NCT03338920) to examine the safety and effectiveness of this intranasal powder in the management of episodic migraine with or without aura in adolescents. Taken together, using AVP-825 may be a superior option compared to oral ST due to its efficiency, safety, and lower side effects. The findings of clinical trials suggest that AVP-825 will be very valuable in treating migraine across multiple attacks and is possibly less affected by GI symptoms. Overall, more investigations by independent researchers are necessary to confirm the collected information related to AVP-825 [6, 53].

### 4.3. ST and Microemulsions

Microemulsion (ME), as a drug carrier, is a thermodynamically stable, transparent (or translucent) mixture of water, oil, and surfactant, which is frequently combined with other cosurfactants with a droplet size of 10-100 nm. These carriers can be classified as water-in-oil, oil-in-water, or bicontinuous systems associated with their structure and are described as ultralow interfacial tension between water and oil phases. Because of its advantages in prolonged release and targeting drugs to a particular site, researchers paid much attention to ME application as a drug delivery system [60]. ME is a good drug delivery system because of the thermodynamic stability, spontaneous formation, easy preparation, elegant and transparent appearance, higher ability to penetrate the biological membranes, elevated drug loading, enhanced bioavailability, and reduced intra- and interindividual variability in the pharmacokinetics of the drug [61].

In a study, intranasal administration of MEs containing ST and SS showed that the blood/brain uptake rates 30 min following intranasal administration were higher than those attained after IV route, suggesting the efficient transport of the drug after intranasal administration of MEs. The results also confirmed the larger and rapid portion of ST-ME transport which helps to reduce the dose and frequency utilizing the ST and enhances the therapeutic index. Thus, the intranasal delivery of ST-ME developed in this research can play a favorable role in managing acute migraine headaches [62]. In another study, the intranasal mucoadhesive ME was characterized. The results showed that SS nasal absorption was fairly improved. These carriers were designed to convert into a gel in the nasal cavity, which could enhance the residence time and bioavailability of the drug. As a result, mucoadhesive ME may be a helpful method to improve rapid-onset delivery of SS during acute treatment of migraine [63].

Moreover, it was reported that an optimized ST-ME could provide rapid transport of the drug across the nasal mucosa and higher stability in the nasal cavity. The brain/blood uptake ratios at 0.5 h of intranasal ST-ME, SS-ME, and ST solution were 0.50, 0.60, and 0.26, respectively [64]. While using ME helps to maximize the concentration of SS or ST and reduce the administration dose, further investigations with different formulations are necessary to adjust an approved delivery system to be applied in the clinic.

### 4.4. ST and Microspheres

The mucoadhesive microsphere (MP) delivery system of drugs is also known as an attractive concept among different systems, which can control the clearance rate of the drug from the nasal cavity and protect the drug against enzymatic degradation [65]. There are limited investigations to show the efficacy of this system in the delivery of ST. Recently, a new formulation was developed to examine the nasal mucoadhesive SS-MPs. The results exposed that the swelling ability, particle size, and incorporation efficiency of MPs enhanced with the elevation of drug/polymer ratio. It is confirmed that hydroxypropyl methylcellulose-based MPs have enough mucoadhesion ability and no adverse effect on the nasal mucosa. This issue suggests that this formulation might be recommended as a promising intranasal delivery system [66]. This new delivery system may be effective and safe in the management of acute attacks of migraine, but further studies are needed.

### 4.5. ST and Nanoparticles

In the last ten years, nanoparticles have been considered drug delivery systems to enhance drug efficacy or reduce toxicity [67–75]. Combining safe and noninvasive nasal drug delivery systems with novel carriers and formulations has facilitated brain-targeted delivery [10, 76]. Among the different agents, chitosan—approved by the FDA—is nontoxic, biodegradable, and nonimmunogenic component found in nature [77]. Several studies have proven that using chitosan-based NPs can enhance the efficiency of intranasal delivery systems [78]. Using mucoadhesive materials, such as starch and chitosan, can increase the deposition time and absorption of drugs in the nasal cavity employed to overcome the low residence time of drugs in the nasal cavity [28, 29]. In a recent study, ST-loaded chitosan NPs were used to improve the therapeutic effect of this drug. The formulation was optimized via the Taguchi method design. Positive zeta potential and suitable entrapment efficiency were obtained [79]. In one previous study, intranasal ST-loaded chitosan nanoparticles (average size: 306.8 ± 3.9 nm) were used to design novel approaches for migraine therapy. The *in vitro* release of the drug from chitosan nanoparticles was assessed in phosphate buffer saline (pH 5.5) using goat nasal mucosa and reported to be 76.7 ± 1.3% within 28 hours. This finding is clearly associated with the features of chitosan, which is easy to dissolve at low pH. Therefore, it can be well supported in the nasal pH range of 5.5 ± 0.5. The findings suggest that this new approach can be a promising drug delivery system with therapeutic properties for migraine [79]. In a similar study, a novel ST delivery system via chitosan nanoparticles was optimized to directly deposit the drug from the nose to the brain. The findings proved that the polymer concentration positively affected entrapment efficiency (71.69 ± 3.24%). The optimized formula also showed a nonaggregated spherical shape with a size of 73.5 ± 1.25 nm. The brain uptake of ST was improved 2.38-fold more than intravenous ST [80].

Formulation and preliminary investigations of the new micellar nanocarriers for intranasal ST administration showed significantly greater uptake of ST compared to ST solution in rats [81].

## 5. Conclusion

Migraine is a common cause of disability that is related to the increased sensitization of the trigeminovascular system. ST, as an agonist of serotonin receptors, is a well-known medication for the treatment of migraine and possesses antioxidant and anti-inflammatory properties. Although intranasal ST administration is a fast-acting route, the bioavailability of liquid intranasal sprays is low. New delivery systems have been developed to improve the bioavailability and effectiveness of ST after intranasal administration. One FDA-approved system is a breath-powered exhalation delivery system (AVP-825) that was recommended as a promising therapy for migraine headache in clinical studies. Some other delivery systems have been defined to enhance the efficacy of ST, such as microemulsions and microspheres as well as chitosan, gliadin (the major component of wheat gluten), and micellar nanocarriers [82]. For example, gliadin nanoparticles have been generally proposed for GI applications because their protein content is rich in neutral and lipophilic amino acids capable of establishing numerous interactions, particularly in the upper zone of the intestinal mucosa [83]. While the findings of preclinical investigations have shown satisfactory efficacy for these systems, further studies are necessary to confirm their application for the treatment of migraine in the clinical setting.
